# Effects of phosphorus fertilization on microbial carbon use efficiency and soil organic matter decomposition in non-allophanic Andosols

**DOI:** 10.3389/fmicb.2026.1851008

**Published:** 2026-06-25

**Authors:** Wako Koizumi, Timothy J. Clough, Soichi Kojima, Tomoyuki Makino, Soh Sugihara, Yoshitaka Uchida, Toru Hamamoto

**Affiliations:** 1 Graduate School of Agricultural Science, Tohoku University, Sendai, Miyagi, Japan; 2 Faculty of Agriculture and Life Sciences, Lincoln University, Lincoln, New Zealand; 3 Graduate School of Agricultural Science, Tokyo University of Agriculture and Technology, Tokyo, Japan; 4 Research Faculty of Agriculture, Hokkaido University, Sapporo, Hokkaido, Japan

**Keywords:** CO_2_ emission, microbial carbon use efficiency, microbial respiration, non-allophanic Andosol, phosphorus-mining, soil organic matter priming

## Abstract

Phosphorus (P) availability affects soil carbon (C) cycling, including microbial C use efficiency (CUE) and priming effects (PEs). While non-allophanic Andosols are characterized by high organic C content and strong P retention, the effects of different P fertilization regimes on C dynamics in these soils remain poorly understood. In this study, we conducted a 20-day incubation experiment using ^13^C-enriched glucose to investigate how different soil available P levels (Truog P: 157 mg P kg^−1^ and 12 mg P kg^−1^) impacted microbial C dynamics in non-allophanic Andosols from contrasting field management practices. Our results showed that soil organic matter (SOM) priming was associated with P fertilization management, with total primed CO_2_-C emissions remaining low in these soils. In high-P soils, the addition of glucose and nitrogen (N) resulted in negative PEs, whereas in low-P soils, the same treatment stimulated microbial SOM mining, resulting in positive PEs. Additionally, glucose-derived CUE was higher in high-P soils than in low-P soils after 20 days of incubation. These findings suggest that long-term P fertilization influences both substrate-induced microbial assimilation and SOM decomposition, with P limitation potentially promoting SOM mining, which, along with concurrent soil acidity and exchangeable Al toxicity, modulates CUE. This study provides insights into improving C sequestration in non-allophanic Andosols through soil fertility management.

## 1 Introduction

Soil organic C (SOC) plays a central role in the global C cycle, as soil microbes degrade SOC, releasing CO_2_. However, C inputs can also enhance SOC stocks through increases in microbial biomass. However, such C inputs may trigger priming effects (PEs) that accelerate SOC decomposition (Blagodatskaya and Kuzyakov, 2008). Therefore, understanding the differential allocation of C between microbial metabolism of SOM and biomass production is critical for assessing SOC sequestration.

Microbial C use efficiency (CUE), the ratio of C allocated to microbial biomass growth to that respired as CO₂, is positively correlated with SOC levels (Tao et al., 2023). The magnitude of CUE has also been shown to depend on microbial community structure (Maynard et al., 2017) and various abiotic factors, such as soil pH and exchangeable aluminum (Al), which can alter microbial metabolism and C allocation (Jones et al., 2019; Schroeder et al., 2024), clay content (Islam et al., 2023), nutrient availability (Manzoni et al., 2012; Spohn, 2016), and substrate type (Jones et al., 2018). Limited nutrient availability and unbalanced substrate additions may lower CUE by promoting excessive enzyme allocation and respiration, a phenomenon described as overflow metabolism (e.g., Tempest and Neijssel, 1992; Russell and Cook, 1995; Schimel and Weintraub, 2003; Keiblinger et al., 2010). According to Manzoni et al. (2012), CUE is also influenced by nitrogen (N) and phosphorus (P) availability, since microbes require these nutrients to balance anabolic and catabolic processes. P is an essential nutrient for soil microbes, as it is a key component of DNA and RNA and plays a critical role in phospholipid synthesis and energy transfer. Depletion of P resources constrains microbial investment in biomass production, shifting C allocation toward enzyme production, which can lower CUE (Zhai et al., 2022). In addition, limited P availability represents a significant agronomic challenge, as it directly constrains plant productivity and undermines soil health. Microbes compete with plants for P, thereby limiting plant P uptake by reducing plant-available P in P-limited soil (Jiang et al., 2024).

An imbalance of available nutrients leads to microbial soil organic matter (SOM) decomposition and nutrient acquisition, resulting in positive priming effects (PEs) (Blagodatskaya and Kuzyakov, 2008; Nottingham et al., 2015; Bernard et al., 2022). For example, the addition of a high C: N substrate induces microbial N acquisition from SOM, a process known as ‘N mining’ (i.e., microbial SOM mineralization induced by N limitation), and positive PEs are observed when compared to the addition of low C: N substrates (Craine et al., 2007; Xu et al., 2024). Immobilization and nutrient mining also apply to P, with high C: P substrates inducing “P-mining” (i.e., microbial SOM mineralization induced by P limitation) and positive PEs under P-limited soil conditions. Therefore, adding N together with the C substrate ensures sufficient N availability to prevent N-mining, thereby isolating P availability as the primary factor driving the observed microbial responses. This allows for the evaluation of how P limitation and P-mining regulate PEs, independent of N-driven SOM decomposition. In some cases, this may also involve the mobilization of P compounds from mineral phases through mechanisms such as the action of competing ligands, protons, or electron-donating compounds (Spohn, 2024; Lieberman et al., 2025). Additionally, the addition of P to soils may contribute to SOM destabilization through abiotic processes. Inputs of P promote the desorption of the organic C adsorbed to mineral surfaces by ligand exchange or the dissolution of organic matter formed as complexes with metals such as Al and iron (Fe) (Kaiser and Zech, 1999; Li et al., 2020; Spohn et al., 2022; Mori, 2023). Previous studies have indicated that P addition increases the bioavailable C pool and C mineralization, contributing to the SOM decomposition in various soils, including Andosols (Miyazawa et al., 2013; Scott et al., 2015; Spohn and Schleuss, 2019; Li et al., 2020).

Considerable research interest has recently focused on P-induced PEs and CUE, and an increasing number of studies examining the effects of P availability on PEs have been conducted using direct P substrate addition, with contrasting results: some showing higher positive PEs but higher CUE (Wang et al., 2025), others showing negative PEs (Wang et al., 2014; Sawada et al., 2023) or negative PEs with high CUE (Zeng et al., 2025), or with higher positive PEs with no effects on CUE (Mehnaz et al., 2019). However, while soils from long-term fertilization trials capture sustained microbial and abiotic responses, they also introduce confounding variables, such as shifts in soil pH and exchangeable Al toxicity, which are inherently associated with P management, particularly in non-allophanic Andosols. Studies using this approach have reported contrasting results. Some studies have observed increased CUE and no significant changes in SOC levels with sustained P input (Li S. et al., 2025), while other studies have found enhanced SOM mineralization and positive PEs (Li et al., 2023; Qin et al., 2024). Moreover, Widdig et al. (2020) reported that P fertilization had no effect on CUE. These varied findings emphasize that microbial responses to P availability are highly context-dependent (e.g., the microbial nutrient mining hypothesis and the microbial stoichiometric decomposition hypothesis; Hessen et al., 2004; Craine et al., 2007). Consequently, the microbial responses observed in these soils reflect the integrated effects of long-term interactions between P availability and associated soil chemical changes rather than the impact of P status alone. Understanding these complex interactions is nonetheless essential for a comprehensive assessment of C cycling under established agricultural management practices. Despite growing interest in this area, a critical knowledge gap remains regarding how P availability, in conjunction with related soil chemical properties, influences microbial CUE and PEs across different soil types and in response to varying substrate quality and quantity, particularly in soils with unique physicochemical properties such as Andosols.

Derived from volcanic ash, Andosols are important for soil C sequestration due to their abundance of Al-organic matter complexes, which contribute to high SOM stability (Nanzyo, 2002; Takahashi and Dahlgren, 2016). Andosols are classified according to their significant P retention and are primarily divided into two groups: allophanic and non-allophanic, which are distinguished by the active Al composition (Takahashi and Dahlgren, 2016; IUSS Working Group WRB, 2022). Non-allophanic Andosols, dominated by Al-organic matter complexes and accounting for approximately 30% of Japanese arable Andosols (Saigusa and Matsuyama, 1998), are characterized by high acidity and high P sorption, with elevated Al phytotoxicity (Shoji et al., 1987; Ito et al., 2011). Given the dependence of soil microbial activity on P availability, it is expected that microbial CUE is affected by P application. However, it remains unknown how CUE responds to long-term P addition in non-allophanic Andosols and how P-induced SOM changes interact with microbial C cycling in these soils.

In this study, we conducted an incubation experiment to investigate the effect of long-term P fertilization on microbial C utilization in non-allophanic Andosols. We used ^13^C glucose to evaluate the impact of P availability on CUE and soil ^13^C recovery. We hypothesized that, (i) in unfertilized soils, substrate additions would promote P-mining, accelerating the positive PEs and (ii) higher CUE would be observed in P-fertilized soils, as the nutrient balance would better match microbial requirements compared to unfertilized soils.

## 2 Materials and methods

### 2.1 Soil sampling

Soil samples were collected at 0–10 cm depth in April 2023 from two contrasting fertilization fields at the Field Science Center, Graduate School of Agricultural Science, Tohoku University, Japan (38°74′N, 140°75′E, 190 m above mean sea level). The soils were classified as non-allophanic Andosols according to the Soil Classification System of Japan (Obara et al., 2011), which corresponds to Aluandic Andosols in the World Reference Base for Soil Resources (IUSS Working Group WRB, 2022). The parent material is dacitic and consists of repeated ash falls that reflect intermittent volcanic activity (Yamada et al., 1980; Shoji et al., 1985). Two contrasting soils with different P content were sampled. The high-P (HP) soil has been under arable land use since at least 1964 and has been managed as an experimental plot with regular P fertilization since 1987. The amount of P input from triple superphosphate and fused magnesium phosphate varied depending on the cultivation years, ranging from 31 to 361 kg P ha^−1^ yr.^−1^, with cattle manure applied infrequently in certain years. In contrast, the low-P (LP) soil, which was converted to agricultural land in 2009 and has never received P fertilization, exhibited a significantly lower Truog P content (12 mg P kg^−1^ soil) than the HP soil (157 mg P kg^−1^ soil). Detailed fertilization and cultivation histories are described in Supplementary Table S1. Physical and chemical properties of the sampled soils are summarized in Table 1 and Supplementary Table S2. Based on particle size distribution, both the HP and LP soils had a clay loam texture. The total C and N contents were 88.1 g C kg^−1^ soil and 5.2 g N kg^−1^ for the HP soil and 91.2 g C kg^−1^ soil and 5.4 g N kg^−1^ soil for the LP soil, respectively. The HP soil (pH 5.29) had a significantly higher pH than the LP soil (pH 4.49). Correspondingly, the LP soil showed a significantly higher amount of exchangeable Al (21.30 meq kg^−1^) than the HP soil (0 meq kg^−1^). The collected soils were sieved through a 2-mm sieve, all visible roots were removed, and the soils were then stored at 4 °C until further use.

**Table 1:** Chemical and biological properties in HP and LP soils (mean [SD]).

Soil	pH_(CaCl2)_		Total C		Total N		Total P		C:N	MBC		MBP		log(MBC:MBP)	Resin P		Truog P		HCl-P	
			(g C kg^−1^)		(g N kg^−1^)		(g P kg^−1^)			(mg C kg^−1^)		(mg P kg^−1^)		Molar basis	(mg P kg^−1^)		(mg P kg^−1^)		(g P kg^−1^)	
HP	5.29	(0.03)	88.07	(3.38)	5.23	(0.23)	2.35	(0.27)	16.83	1368.71	(56.55)	75.06	(18.31)	1.68	122.39	(6.31)	157.31	(5.07)	1.41	(0.029)
LP	4.49	(0.06)	91.24	(1.99)	5.36	(0.10)	1.37	(0.14)	17.01	1343.65	(142.09)	21.31	(11.63)	2.31	29.13	(1.33)	12.10	(0.31)	0.54	(0.063)
*p*-values	**<0.0001**		0.17		0.35		**<0.01**		**<0.05**	0.76		**<0.01**		**<0.05**	**<0.001**		**<0.0001**		**<0.0001**	

For each property, significant differences between HP and LP soils were determined by independent samples *t*-tests.

MBC, microbial biomass C; MBP, microbial biomass P; MBC: MBP, their molar-based ratio; resin P, resin-extractable P. Truog P and HCl-P represent Truog-extractable P and 1M HCl-extractable P, respectively.

Bold values indicate statistically significant p-values at *p* < 0.05.

### 2.2 Experimental design

Prior to the experiment, the sieved soils were pre-incubated in the dark at 25 °C for 14 days and maintained at a gravimetric moisture content of 45% (w/w). Pre-incubated soils were placed in plastic bags (each equivalent to 100 g dry weight). Soil samples received a substrate solution containing either (1) water (“Control”), (2) ^13^C-enriched glucose (“glc”), or (3) ^13^C-enriched glucose and (NH_4_)_2_SO_4_ (“glcN”). There were 24 samples in total (two soils × three glucose levels × four replicates). The rate of added ^13^C-enriched glucose (4 atom%, D-GLUCOSE (U^−13^C6, 99%), Cambridge, 450 mg C kg^−1^ soil) was set to be 25–50% of the microbial biomass C (MBC) to increase microbial activity while avoiding exponential microbial growth and shifts in microbial community composition (Blagodatskaya and Kuzyakov, 2008; Sawada et al., 2023). A total of 48 microcosms were prepared (2 soil types × 3 substrate treatments × 4 replicates × 2 sampling days). To evaluate temporal dynamics, half of the microcosms (*n* = 24) were destructively sampled on day 2. The remaining 24 microcosms were incubated for 20 days. These 20-day microcosms were used to measure soil respiration at six intervals (days 1, 2, 6, 8, 12, and 20) prior to being destructively sampled on day 20 to determine microbial biomass and other soil parameters. The concentration of added mineral N [61 mg N kg^−1^ soil, supplied as (NH₄)₂SO₄] was determined according to the mean microbial C: N: P ratios of 60:7:1 (Cleveland and Liptzin, 2007). The experiment was then started by adjusting the soil to 55% gravimetric moisture content using each substrate solution.

### 2.3 Analysis of soil sample

#### 2.3.1 CO_2_ emission

For the measurement of soil respiration, each treatment was placed in a 450-mL airtight glass jar with a CO_2_ alkali trap (5 mL of 1 M NaOH) and incubated at 25 °C for 20 days. Soil respiration was measured at days 1, 2, 6, 8, 12, and 20 of incubation. A volume of 10 mL of 0.01 M HCl solution was also placed in the glass jar to maintain soil moisture conditions. Blank jars without soil were also prepared and incubated under the same conditions. The blank values were subtracted from the sample measurements to correct for background CO₂ in all subsequent calculations. The collected NaOH solutions received 8 mL of 1 M SrCl_2_ to form SrCO_3_ precipitate, and the resulting mixture of precipitate and solution was titrated using a 1 M HCl solution (Zibilske, 1994; Qiao et al., 2014). After titrating, the precipitates were washed three times with 30 mL of water and then dried at 105 °C. The dried SrCO_3_ was packed in tin capsules to analyze for ^13^C enrichment using a DELTA V plus isotope-ratio mass spectrometer (IRMS, Thermo Fisher Scientific, Bremen, Germany). The calculation of glucose-derived CO_2_-C_glc_ was adapted from the method of Mehnaz et al. (2019) and Brant et al. (2006) (Equation 1):


$${\mathrm{CO}}_{2}-C_{\mathrm{glc}}={\mathrm{CO}}_{2}-C\times (C13_{c}–C13_{\text{none}})/(C13_{\text{label}}–C13_{\text{none}})$$ (1)

where CO_2_-C is the cumulative CO_2_-C respired from the soil with glucose addition, and ^13^C_c_, ^13^C_none_, and ^13^C labels are the ^13^C atom% values of the CO_2_-C measured in the NaOH solutions with and without glucose addition and the added glucose substrate, respectively. Soil-derived CO_2_-C (CO_2_-C_soil_) in glc and glcN treatments was calculated as the difference between the total (CO_2_-C) and glucose-derived CO_2_-C_glc_.

#### 2.3.2 Soil microbial biomass C and P

Soil microbial biomass C (MBC) was determined using a modified chloroform-fumigated method (Beck et al., 1997). Briefly, two samples of 5 g dry soil for each sample, with and without 24 h fumigation using chloroform, were extracted with 25 mL of 0.05 M K_2_SO_4_ and were analyzed for total organic C using a TOC-TN analyzer (TOC-V CPH/CPN, Shimadzu, Japan). The MBC was calculated from the difference in total organic C concentrations in samples with and without fumigation, divided by an extraction efficiency of 0.45 (Beck et al., 1997). The ^13^C atom% in each extract was measured on an IRMS (Thermo Fisher Scientific, United States). The ^13^C atom% of the microbial biomass (^13^C_mic_) and the glucose-derived C in the microbial biomass (MBC_glucose_) were also calculated by the following Equations 2, 3 (Mehnaz et al., 2019):


$$C13_{\mathrm{mic}}=(C_{f}\times C13_{f}-C_{\mathrm{nf}}\times C13_{\mathrm{nf}})/(C_{f}-C_{\mathrm{nf}})$$ (2)

$${\mathrm{MBC}}_{\text{glucose}}=\mathrm{MBC}\times (C13_{\mathrm{mic},c}-C13_{\mathrm{mic},\text{none}})/(C_{f}-C_{\mathrm{nf}})$$ (3)

where *C_f_* and ^13^C_f_ are the amounts of C and ^13^C atom% in the fumigated extracts and C_nf_ and ^13^C_nf_ are those in the non-fumigated extracts, respectively. Organic C within K_2_SO_4_ extracts from non-fumigated soil was denoted as dissolved organic C (DOC). The terms ^13^C_mic,c_ and ^13^C_mic,none_ are the ^13^C_mic_ values with and without glc addition.

Soil microbial biomass P (MBP) was measured using an anion exchange membrane method according to Kouno et al. (1995). Briefly, soil samples (1 g dry mass equivalent) were shaken and extracted in a 20-mL mixture of deionized water and resin for 16 h. For fumigated samples only, 2 mL of hexanol was added instead of chloroform, which was used in the method of Kouno et al. (1995). The resin was then removed, and the phosphate was recovered by shaking for 1 h in 20 mL of 0.5 M HCl. We added 25 and 40 mg P kg^−1,^ depending on P contents as a spike, considering the P adsorption in LP and HP soils, respectively (Brookes et al., 1982). The extracted P was determined using the malachite green colorimetric method (Ohno and Zibilske, 1991; Jeannotte et al., 2004; Song et al., 2019). The extracts were diluted appropriately to avoid interference from HCl (D’Angelo et al., 2001) and analyzed by absorbance measurement at 630 nm using a microplate reader (iMark, Bio-Rad Laboratories, United States). The MBP was calculated from the difference in extracted P concentrations between samples with and without fumigation, divided by 0.40 (Brookes et al., 1982). Both MBC and MBP were measured at days 2 and 20 of incubation.

#### 2.3.3 Soil P measurements

Phosphate within non-applied hexanol extracts for analysis of MBP was denoted as resin-extractable P (resin P). Resin P represents a part of labile P pools (Erinle et al., 2020) and avoids P fixation by soil colloids during extraction in Andosols (Kouno et al., 1995). However, we note that accurately assessing P availability in Andosols with high P sorption capacity using a single chemical extraction method is difficult (Fixen and Grove, 1990; Sugito and Shinano, 2013). The 1 M HCl-extractable P described by DeLuca et al. (2015) was also measured. It primarily represents P mobilized by protons released by microbes or plants, primarily from apatite and other recalcitrant inorganic P forms, and also partially extracts Al/Fe-bound P and phosphate, which are weakly adsorbed to clay particles or present in inorganic precipitates (Kuo, 1996; Pingree et al., 2017; Wuenscher et al., 2015; Li et al., 2019). This fraction was denoted as HCl-P. It was measured by extracting 0.5 g of dry mass equivalent soil with 10 mL of 1 M HCl for 3 h at 200 rev min^−1^ at room temperature. After filtering, the inorganic P was determined using the malachite green method at 630 nm with a microplate reader (iMark, Bio-Rad Laboratories, United States) for the MBP analysis.

#### 2.3.4 Inorganic N measurements

Inorganic N (IN) was determined as the sum of NH₄^+^-N and NO₃^−^-N. Soil samples (5 g dry mass equivalent) from the 2- and 20-day incubation periods were extracted with 25 mL of 1 M KCl by shaking for 30 min. The concentrations of NH₄^+^-N and NO₃^−^-N in the filtrates were analyzed using a flow injection analyzer (AQLA-700, Aqualab Co., Ltd., Japan), with NH₄^+^-N measured by absorbance at 630 nm and NO₃^−^-N by absorbance at 540 nm, as described by Hamamoto and Uchida (2015).

#### 2.3.5 Substrate-derived C use efficiency and priming effects

The calculations of glucose-derived CUE and PEs were adapted from the study by Mehnaz et al. (2019) (Equation 4). While the CUE measured in this study strictly represents glucose use efficiency, we used the term “glucose-derived CUE” to maintain consistency with similar previous studies (e.g., Cui et al., 2022; Karhu et al., 2022; Kalu et al., 2024; Xiao et al., 2021). Microbial glucose-derived CUE (CUE_glucose_) of the added glucose was calculated using the following equation:


$${\mathrm{CUE}}_{\text{glucose}}={\mathrm{MBC}}_{\text{glucose}}/({\mathrm{MBC}}_{\text{glucose}}+{\mathrm{CO}}_{2}-C_{\text{glucose}})$$ (4)

The PEs were calculated as the increase in native soil organic C released after substrate application using the following Equation 5 (Mehnaz et al., 2019):


$$\mathrm{PEs}={\mathrm{CO}}_{2}-C_{\text{soil}}-{\mathrm{CO}}_{2}-C_{\mathrm{ctr}}$$ (5)

where CO_2_-C_soil_ and CO_2_-C_ctr_ are the soil-derived CO_2_-C respired from the soil with glucose addition and the cumulative CO_2_-C respired from the soil without glucose addition, respectively.

#### 2.3.6 Soil extracellular enzyme activities

The potential activity of acid phosphomonoesterase (EC 3.1.3.2), P-acquiring extracellular enzyme activity (EEA), was measured fluorometrically with methylumbelliferone (Bell et al., 2013). Briefly, soil samples (1.5 g dry mass equivalent) with 45 mL of deionized water were shaken for 30 min (Deng et al., 2011; Li C. et al., 2025). A volume of 800 μL of mixed soil slurry was transferred into a deep-well plate, and 200 μL of 1 mM substrate (4-methylumbelliferyl phosphate) was then added and incubated at 35 °C for 1.5 h. After incubation, the samples were centrifuged for 3 min at 2,500 × g and transferred to the 96-well black microplate. Fluorescence intensity was measured using a Varioskan ALF multi-mode microplate reader (Thermo Scientific, United States) at 345 nm excitation and 450 nm emission. EEA was expressed as nmol g^−1^ h^−1^.

### 2.4 Statistical analysis

All statistical analyses were performed using R (version 4.2.2; R Core Team, 2023). We conducted a two-way analysis of variance (ANOVA), including the interaction between soils and substrates, to assess their effects on the total CO_2_-C, glucose-derived CO_2_-C, and soil-derived CO_2_-C using a linear model (Bates et al., 2015). We also conducted a three-way ANOVA, including the interaction between soils and substrates, soils and time, and substrates and time, to assess their effects on total MBC, glucose-derived and soil-derived MBC, DOC, MBP, HCl-P, resin P, and EEA using a linear model. Prior to the analysis, the datasets were rank-transformed to meet the assumptions of ANOVA. The model was validated by examining residuals using the DHARMa package (Hartig, 2016). The data were further analyzed using Tukey’s honestly significant difference (HSD) test to determine pairwise differences between soil type and substrate combinations at α = 0.05 using the *multcomp* package (Hothorn et al., 2008). The effects of soil P fertilization on each result and the addition of N on PEs were determined using independent-samples *t*-tests. For the PEs, a repeated measures ANOVA was also performed to evaluate the overall effects of soil and substrate across the entire incubation period.

## 3 Results

### 3.1 CO_2_ emission

**Figure 1:**
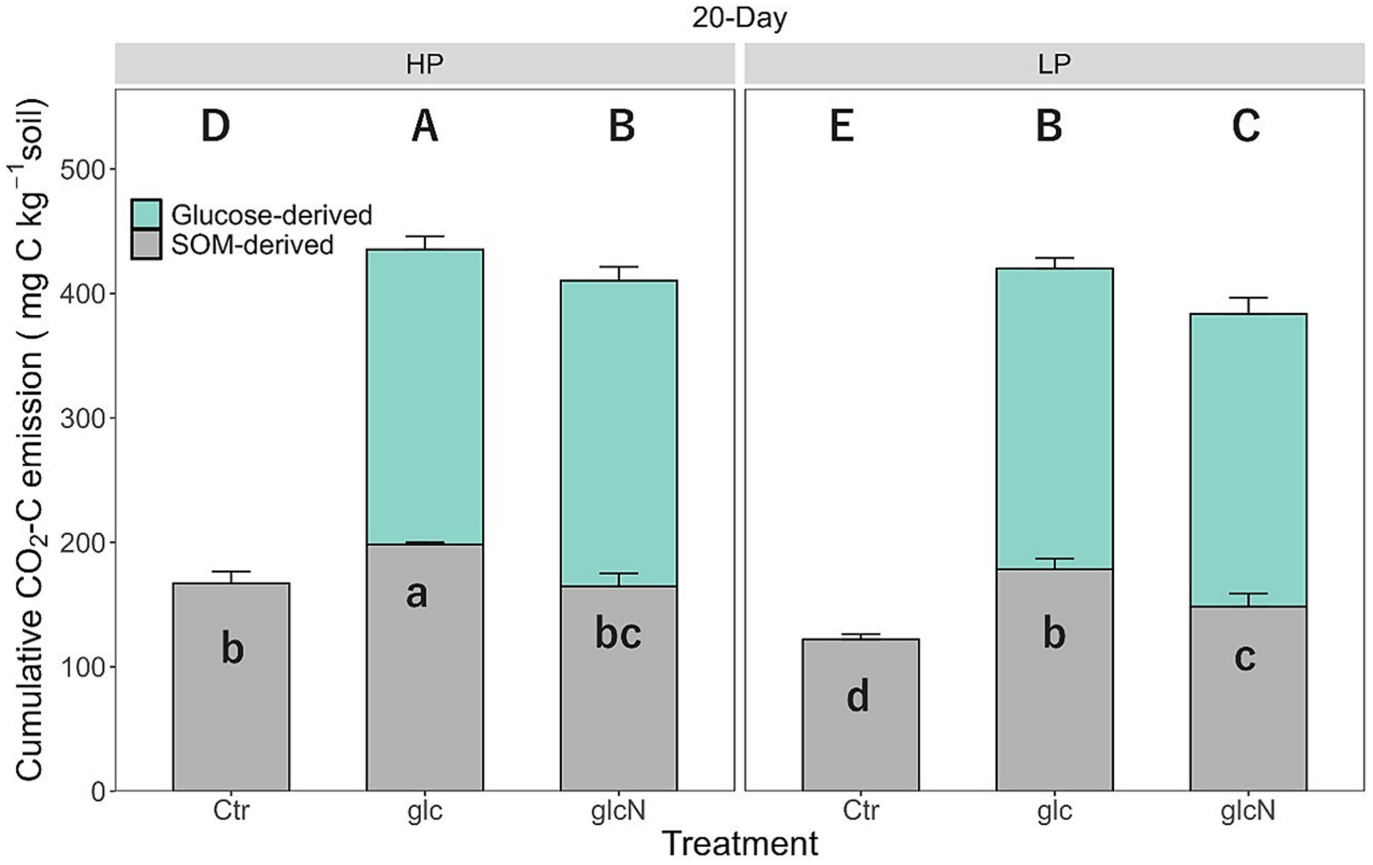
Cumulative CO_2_ emissions (mg C kg^−1^ soil) over 20-day incubation. Treatments include soil without any amendment (Ctr), soil with ^13^C-enriched glucose (glc), and soil with ^13^C-enriched glucose and N substrates (glcN). Different color bars show cumulative CO_2_ emissions (mean ± SD, *n* = 4, but *n* = 3 in LP Ctr due to an operational error during the experiment) derived from added glucose (light green) and soil organic matter (gray). Uppercase and lowercase letters indicate significant differences (*p* < 0.05) in total and SOM-derived CO_2_-C among treatments, respectively.

The HP soil had a higher cumulative CO_2_ emission (339.3 mg C kg^−1^ soil) than the LP soil (308.6 mg C kg^−1^ soil), representing an approximately 10% increase when averaged across substrate treatments during the incubation period (Figure 1; Supplementary Table S1). As no significant interaction was detected between soil type and substrate treatment for cumulative CO₂ emissions (Supplementary Table S3), the main effects of each factor were interpreted independently. Substrate additions significantly stimulated CO_2_ emissions from both soils at day 1, immediately after the incubation (Supplementary Figure S1; Supplementary Table S3). This initial increase was primarily due to the glucose-derived CO_2_ emission, which peaked at 161.2 mg C kg^−1^ in the HP soil and 154.5 mg C kg^−1^ in the LP soil across substrate treatments (Supplementary Figure S1). Among substrate treatments, the glc treatments had significantly higher cumulative CO_2_ emissions than the glcN treatments in each soil (Figure 1; Supplementary Table S3).

The cumulative SOM-derived CO₂ emissions were significantly higher in the HP soil than in the LP soil across all treatments (*p* < 0.0001, Figure 1; Supplementary Table S3). In the control treatment, SOM-derived CO₂ emissions were 167 and 122 mg C kg^−1^ soil in the HP and LP soils, respectively. Substrate addition significantly increased SOM-derived CO₂ emissions in both soils from day 6 after the start of incubation (*p <* 0.001, Supplementary Table S3). In the HP soil, the glc treatment increased SOM-derived cumulative CO₂ emissions by 36 mg C kg^−1^ soil compared to the control, whereas in the LP soil, both the glc and glcN treatments significantly increased these emissions by 55 and 21 mg C kg^−1^ soil, respectively.

In contrast to the total and SOM-derived CO_2_ emissions, the cumulative glucose-derived CO_2_ emission showed no significant differences among soils and substrate treatments throughout the incubation period (Supplementary Figure S2).

**Figure 2:**
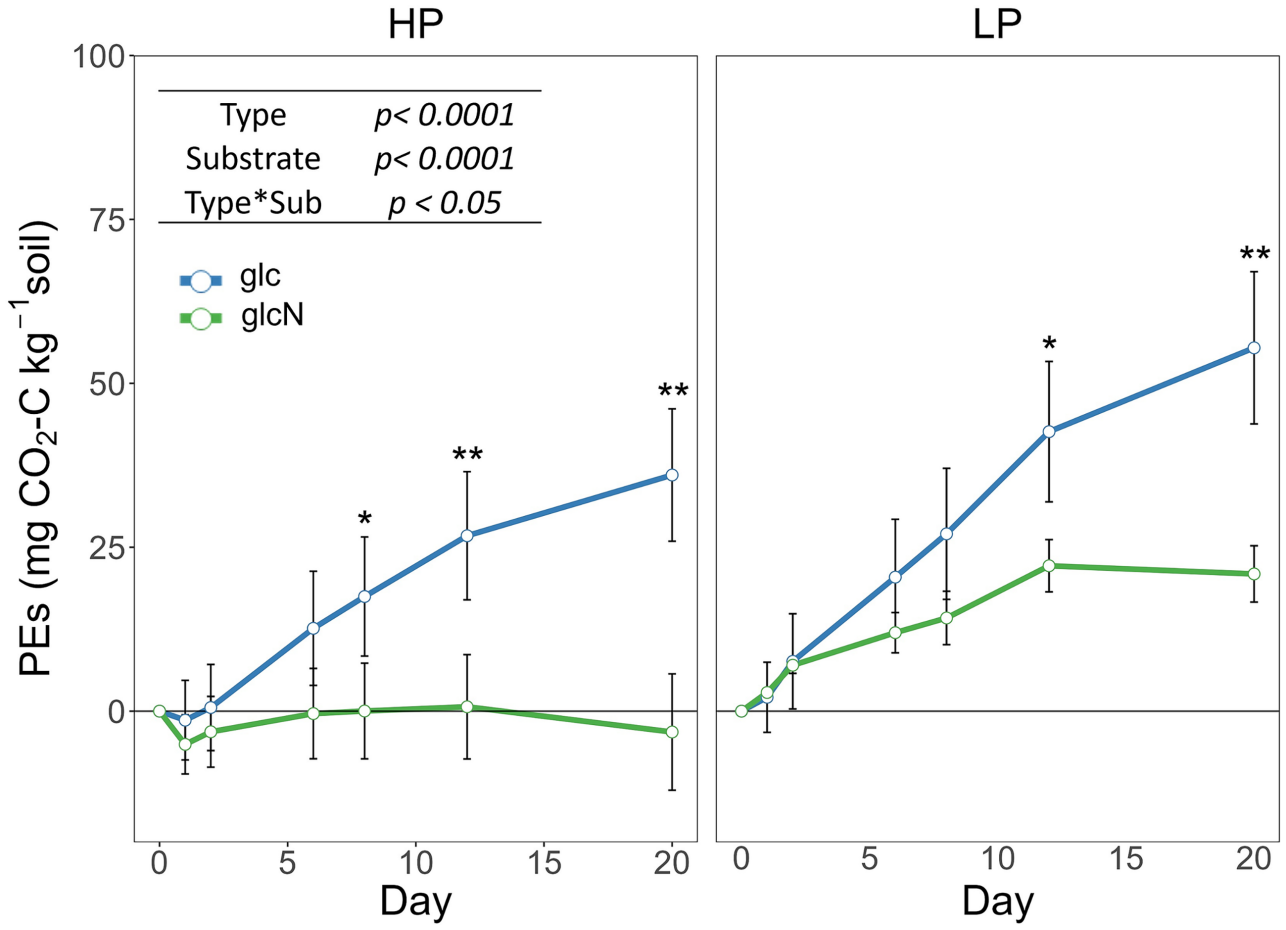
The priming effects (PEs) in HP and LP soils over 20 days. Treatments include soil with ^13^C-enriched glucose (glc) and soil with ^13^C-enriched glucose and N substrates (glcN). Green and blue lines show the priming effect (mean ± SD, *n* = 4) in glc and glcN, respectively, for each soil. * (*p* < 0.05), ** (*p* < 0.01), and blank (not significant) indicate the significance level of the N addition effect for each soil separately. Two-way ANOVA results at each individual measurement interval are presented in Supplementary Table S3. Furthermore, a repeated measures ANOVA was performed to evaluate the overall effects of soil type (HP vs. LP) and substrate treatment (glc vs. glcN), as well as their interaction (Type × Substrate), across the entire incubation period, with the results presented in the table in the upper left.

The PEs during incubation were consistently lower in the HP soil than in the LP soil across the treatments (Figure 2; Supplementary Tables S3, S4). The difference in PE between LP and HP soils within the same substrate treatment ranged from 3.49 mg C kg^−1^ soil at day 1 to 24.11 mg C kg^−1^ soil at day 20. The addition of glc had significantly higher PEs than the addition of glcN in both soils. The addition of glc and glcN in the LP soil resulted in positive PEs. However, the effect of substrate treatments on PEs in the HP soil contrasted with the LP soil; the addition of glc resulted in positive PEs; however, the addition of glcN resulted in negative PEs. The total primed CO_2_-C ranged from −0.71 to 12.88% of the added glucose C. When expressed in terms of the total soil C content, these primed values accounted for −0.008‰ to 0.135‰ (Supplementary Table S4).

### 3.2 Microbial biomass, soil chemical properties, and soil enzyme activity

**Figure 3:**
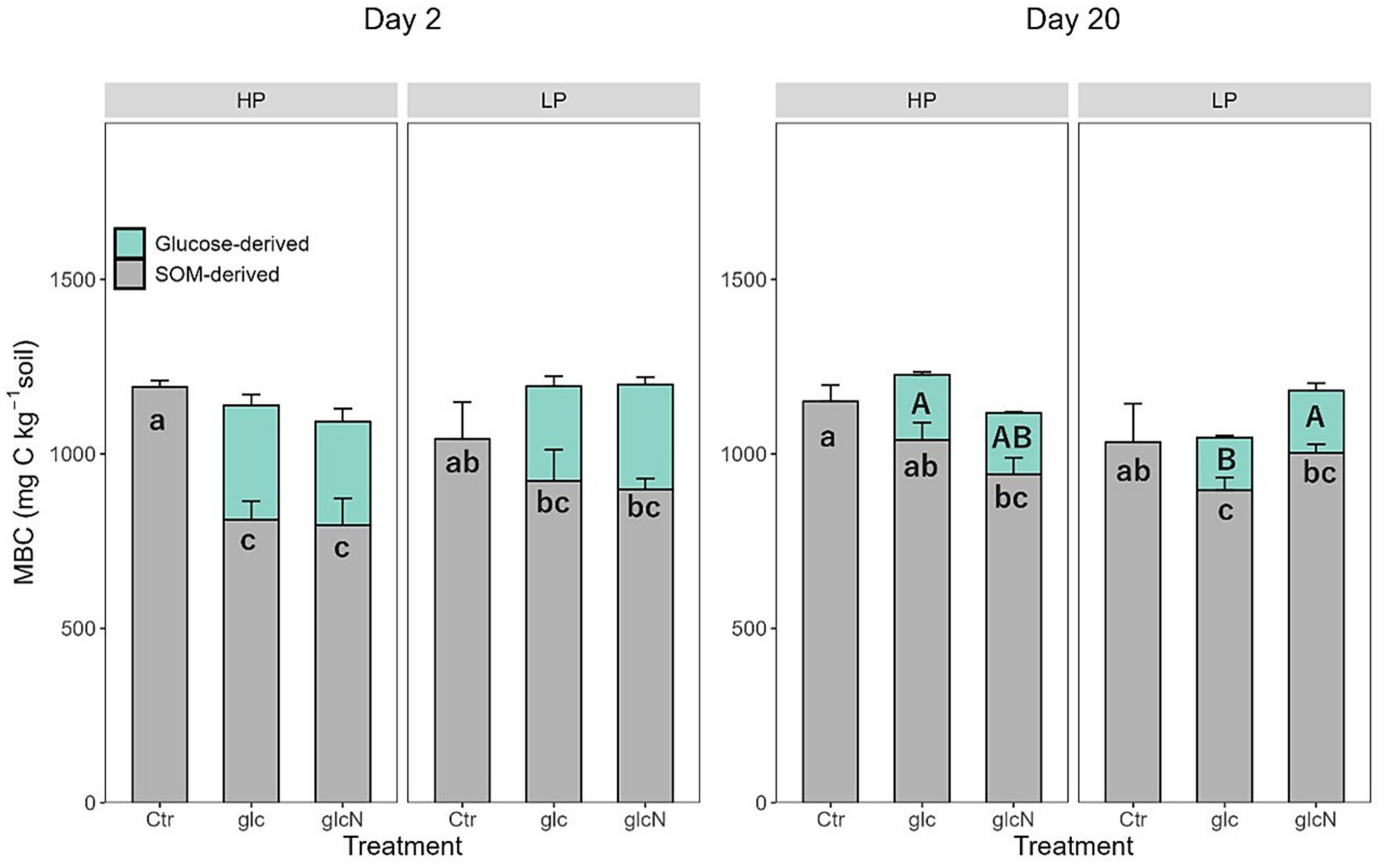
Microbial biomass C (MBC) (mg C kg^−1^ soil) in 2- and 20-day incubations. Treatments include soil without any amendment (control), soil with ^13^C-enriched glucose (glc), and soil with ^13^C-enriched glucose and N substrates (glcN). Different color bars show cumulative MBC (mean ± SD, *n* = 4, but *n* = 3 in LP Ctr due to an operational error during the experiment) derived from the added glucose (light green) and soil organic matter (gray). Uppercase and lowercase letters indicate significant differences (*p* < 0.05) in glucose-derived MBC and SOM-derived MBC among substrate treatments and soils at each sampling time, respectively.

While no significant main effect of soil or substrate was observed for MBC_Total_, there was a significant three-way interaction between soil, substrate, and day (*p* < 0.05, Table 2). Specifically, in the glc treatment, the HP soil had significantly higher MBC_Total_ than the LP soil at 20 days of incubation (*p* < 0.05; Figure 3; Table 2), with a difference of approximately 180 mg C kg^−1^ soil between the two soils, whereas no such differences were observed after 2 days of incubation. Substrate additions did not affect MBC_Total_ in either soil at either 2 or 20 days of incubation (Table 2). For the SOM-derived microbial biomass C (MBC_SOM_), substrate additions significantly decreased MBC_SOM_ in the HP soil compared to the control at 2 days of incubation (Figure 3; Table 2). In contrast, the LP soil showed no differences in MBC_SOM_ among substrate treatments at 2 days of incubation. Glucose-derived microbial biomass C (MBC_Glc_) showed no significant differences among soils and substrate treatments at 2 days of incubation (Figure 3; Table 2). However, at 20 days of incubation, MBC_Glc_ was higher in the HP soil than in the LP soil. In both soils, MBC_Glc_ was significantly lower at 20 days of incubation than at 2 days. The P-acquiring enzyme activity was higher in HP soils than in LP soils (Table 2). The addition of glcN significantly increased the P-acquiring enzyme activity in LP soils at 2 and 20 days of incubation.

**Table 2:** Microbial biomass nutrients and statistical significances at 2 and 20 days after incubation (mean ± SD, *n* = 4, but *n* = 3 in LP Ctr at 20 days and in LP glc (MBP and log(MBC:MBP)) at 2 days due to an operational error during the experiment).

Treatment		MBC_total_					MBC_glucose-derived_					MBC_SOM-derived_						MBP					log(MBC:MBP)			PHOS					
		(mg C kg^−1^)					(mg C kg^−1^)					(mg C kg^−1^)						(mg P kg^−1^)					molar basis			(umol g^−1^ h^−1^)					
Soil	Substrate	2-day		20-day			2-day		20-day			2-day			20-day			2-day		20-day			2-day	20-day		2-day			20-day		
HP	Ctr	1,192	(17.87)	1,150	(40.88)	ab	–		–			1192.43	(17.87)	a	1150.40	(46.66)	a	75.12	(44.63)	89.53	(34.77)	a	1.69	1.56	c	2.31	(0.057)	abc	2.30	(0.041)	b
	glc	1,139	(78.65)	1,227	(53.34)	a	327.30	(31.86)	187.28	(8.75)	a	811.56	(53.31)	c	1039.62	(50.13)	ab	31.93	(9.46)	44.49	(18.75)	ab	1.98	1.90	bc	2.32	(0.048)	ab	2.25	(0.024)	b
	glcN	1,092	(112.09)	1,117	(43.25)	ab	297.04	(37.23)	175.55	(3.02)	ab	795.25	(76.91)	c	941.91	(46.95)	bc	56.14	(47.52)	105.80	(69.78)	a	1.98	1.51	c	2.49	(0.044)	a	2.54	(0.060)	a
LP	Ctr	1,042	(106.56)	1,033	(88.50)	ab	–		–			1042.36	(106.56)	ab	1033.33	(110.38)	ab	29.71	(7.39)	21.23	(6.85)	ab	1.97	2.12	ab	2.02	(0.202)	c	1.91	(0.064)	d
	glc	1,194	(116.29)	1,047	(36.55)	b	271.45	(29.07)	150.61	(5.94)	b	922.48	(89.54)	bc	896.21	(36.43)	c	24.84	(16.00)	17.75	(3.05)	b	2.18	2.19	ab	2.21	(0.098)	bc	2.02	(0.050)	cd
	glcN	1,199	(52.20)	1,181	(28.09)	ab	300.43	(21.27)	178.50	(21.49)	ab	898.57	(31.12)	c	1002.68	(24.59)	bc	23.01	(13.11)	13.24	(8.82)	b	2.23	2.50	a	2.41	(0.127)	ab	2.18	(0.087)	bc
ANOVA *p*-values																															
Soil		0.25					**<0.05**					0.25						**<0.0001**					**<0.0001**			**<0.0001**					
Substrate		0.33					0.61					**<0.0001**						0.092					0.06			**<0.0001**					
Day		0.34					**<0.0001**					**<0.001**						0.92					0.91			**<0.001**					
Soil × Sub		**<0.01**					**<0.01**					**<0.001**						0.59					0.46			0.21					
Soil × Day		0.060					0.80					**<0.05**						**<0.01**					**<0.05**			**<0.05**					
Sub × Day		0.90					0.49					**<0.01**						0.86					0.86			0.44					
Soil × Sub × Day		**<0.05**					0.55					**<0.001**						0.73					0.70			0.15					

Levels of significance were based on a three-way analysis of variance (ANOVA). Different letters indicate significant differences according to Tukey’s HSD *post-hoc* test.

MBC glucose-derived, microbial biomass C derived from glucose; MBC SOM-derived, microbial biomass C derived from SOM; MBP, microbial biomass P; MBC:MBP is their molar-based ratio; PHOS, microbial P-acquired enzyme activity.

Bold values indicate statistically significant p-values at *p* < 0.05.

The HP soil had significantly higher MBP and a lower log ratio of MBC: MBP than the LP soil at 2 and 20 days of incubation (Tables 1, 2). Although the main effect of day was not significant, a significant soil × day interaction was observed for both MBP (*p* < 0.01) and MBC: MBP (*p* < 0.05) (Table 2), reflecting that the magnitude of difference between HP and LP soils became increasingly pronounced from day 2 to day 20. MBP in the HP soil averaged 80 mg P kg^−1^ soil, approximately 4.6 times higher than that in the LP soil (17 mg P kg^−1^ soil). The largest effect size was observed in the glcN treatment, where MBP in the HP soil was approximately 8-fold higher than in the LP soil at 2 days of incubation. However, substrate additions had no significant effect on MBP in both soils during the incubation period.

Soil pH, resin P, and HCl-P were consistently higher in the HP soils than in the LP soils during the incubation period (Supplementary Table S5). Resin P increased from 0 to 2 days of incubation in the HP soil (*p* < 0.001), whereas there was no significant difference in the LP soil (*p* = 0.065). Then, resin P decreased from 2 to 20 days in the LP soil (*p* < 0.001), whereas no significant difference was observed in the HP soil (*p* = 0.41).

### 3.3 Microbial C use efficiency

Glucose-derived microbial C use efficiency (CUE) ranged from 0.61 to 0.65 at 2 days of incubation and 0.39 to 0.44 at 20 days of incubation (Table 3). CUE showed no significant differences between the two soils or substrate additions at 2 days after incubation. However, at 20 days after incubation, glucose-derived CUE was higher in the HP soil than in the LP soil, representing an approximately 0.02 increase when averaged across substrate treatments (*p* < 0.05). The HP soil with glc showed the highest glucose use efficiency, whereas the LP soil with glc had the lowest glucose use efficiency at 20 days after incubation.

**Table 3:** C use efficiency (CUE) in the HP and LP soils at 2 and 20 days after incubation (mean [SD]).

	CUE					
Soil	Substrate	2-day		20-day		
HP	glc	0.65	(0.025)	0.44	(0.015)	a
	glcN	0.61	(0.038)	0.42	(0.009)	ab
LP	glc	0.62	(0.035)	0.39	(0.012)	b
	glcN	0.63	(0.012)	0.43	(0.025)	a
ANOVA *p*-values						
Soil		0.50		**<0.05**		
Substrate		0.27		0.21		
Soil × sub		0.06		**<0.01**		

Different letters indicate significant differences (*p* < 0.05) among treatments for each soil type.

Bold values indicate statistically significant p-values at *p* < 0.05.

## 4 Discussion

Our results reveal that contrasting long-term P fertilization affected the microbial respiration. The HP soils had significantly higher cumulative CO_2_ emissions in the control than in the LP soils, and this trend also remained consistent regardless of the glc or glcN addition. This finding suggests that P input potentially enhanced the microbial activity due to increasing available P and could also promote the desorption of organic C, thereby increasing available C pools (Kaiser and Zech, 1999; Miyazawa et al., 2013; Scott et al., 2015; Spohn and Schleuss, 2019; Li et al., 2020; Spohn et al., 2022).

PEs were also influenced by P fertilization (Figure 2). The higher SOM-derived CO₂ emissions in the HP soil during the early incubation period (after 2 days) reflected its greater baseline microbial activity compared to the LP soil, where microbial activity was likely suppressed by P limitation, soil acidity, and Al^3+^ toxicity. Nevertheless, the magnitude of the substrate-induced increase in SOM decomposition (i.e., PE) was significantly greater in the LP soil (Figure 2), suggesting that P-limited microbes were more responsive to fresh C input in terms of SOM mineralization driven by P limitation (“P-mining”). Notably, SOM mineralization induced by N limitation (“N-mining”) was suggested in both soils by the higher soil-derived CO₂ emissions with glucose-only addition compared to glucose+N addition (Li et al., 2017). However, the overall response of PEs to substrate addition clearly contrasted with soil P status. Specifically, when N was not limited (i.e., in the glcN treatment), negative PEs were found in the HP soil, whereas positive PEs were observed in the LP soil. Combined with the significantly increased phosphatase activity in the LP soil (Table 2), this result indicates that the positive PEs are associated with SOM degradation driven by P-mining, supporting our first hypothesis. In addition, the LP soils had consistently lower resin P and higher MBC: P ratios than the HP soils (Tables 1, 2; Supplementary Table S5). Under nutrient-imbalanced conditions, microbes invest abundant elements into decomposing SOM to obtain limited nutrients, thereby maximizing their productivity (Allison and Vitousek, 2005; Mooshammer et al., 2014). Moreover, MBP levels in the LP soil remained stable throughout the incubation period, possibly because positive priming induced P-mining (Spohn, 2016).

Regarding microbial biomass dynamics, the lack of increase in MBC_Total_ in the HP soil at day 2 likely reflects a combination of preferential substrate utilization and pool substitution effects (Wang et al., 2026). On day 2, the HP soil showed a negative PE (Figure 2), suggesting that microbes may have switched their activity from native SOM to the added substrate C. As shown in Table 2, while significant glucose-derived C was incorporated into the biomass (MBC_glc_), it was offset by a significant decrease in SOM-derived microbial C (MBC_SOM_). This rapid biomass turnover, coupled with the observed negative PE, suggests that microbes utilized the added substrate instead of decomposing native SOM, leading to a temporary suppression of SOM-derived CO₂ emissions while replacing C within the existing biomass pool.

In agreement with our second hypothesis, P fertilization was also associated with CUE. After 20 days of incubation, the HP soils showed significantly higher glucose-derived CUE than in the LP soil (Figures 1, 3; Table 3). These long-term CUE measurements (>48 h) suggest the potential for C retention through the recycling of microbial necromass and exudates, which can eventually contribute to SOC stabilization (Geyer et al., 2016; Kalu et al., 2024). Although recycled C may not always avoid mineralization, a higher long-term CUE (>48 h) indicates a greater proportion of C being incorporated into microbial products rather than being immediately respired, in contrast to short-term CUE (<48 h), which primarily reflects substrate utilization for gross production (Brant et al., 2006; Geyer et al., 2016). After 20 days of incubation in the LP soil, microbes allocated more energy to catabolic pathways to acquire P, which in turn reduced C available for growth and resulted in a lower glucose-derived CUE than in the HP soil (Bernard et al., 2022). Furthermore, the lack of a significant increase in MBC following N addition compared to the glucose-only treatment (Figure 3; Table 2) indicates that N availability was not the primary constraint on microbial biomass growth in these non-allophanic Andosols. These findings suggest that the difference in long-term P fertilization history between the two soils is a key factor driving the observed variation in CUE and PEs.

The two soils used in this study significantly differed in pH and exchangeable acidity due to long-term P fertilization, both of which are significant factors influencing microbial activity. The lower pH throughout the incubation period and higher exchangeable Al in the LP soils compared to the HP soils likely suppressed microbial respiration and activity (Table 1; Supplementary Table S5). In general, higher positive PEs occur in soils with neutral pH compared to the soils with acidic pH because the microbial activity, the community structure, and the enzyme synthesis are more pronounced (Blagodatskaya and Kuzyakov, 2008; Blagodatskaya and Anderson, 1998). However, a previous study suggested that greater positive PEs observed in strongly acidic soils are associated with the size of labile C pools and a net increase in MBC (Aye et al., 2018). In this study, although MBC showed no significant differences among soils, higher DOC concentrations were observed in the LP soils across the treatments, which corresponded with the greater primed CO_2_ emissions observed (Figure 3; Table 2). These findings indicated a potential role of low pH in affecting DOC pools and microbial resource accessibility. In contrast, MBP was significantly lower in the LP soils than in the HP soils, and as discussed above, the higher MBC: P ratio likely reflects SOM degradation induced by P-mining.

In addition to PEs, soil pH is also important for understanding the CUE results; however, after 2 days of incubation, glucose-derived CUE showed no significant differences among soils (Table 3). It is possible that the effect of P fertilization on microbial assimilation of substrates (i.e., short-term CUE) after 2 days of incubation was offset by (i) availability of organic C and (ii) soil pH, both of which are specific to non-allophanic Andosols. Soil C content is one of the regulating factors of microbial assimilation (Kamble and Bååth, 2014); Andosols have a large amount of organic C content due to Al-soil organic matter complexes (Takahashi and Dahlgren, 2016). Moreover, it has been suggested that non-allophanic Andosols have higher organic C contents than other Andosols (Nanzyo et al., 1993). Previous studies have shown that microbial growth and CUE are strongly related to C availability rather than N and P availability (Wang et al., 2021; Morris et al., 2022; Jiang et al., 2025). In this study, we have found that HP and LP soils contain approximately 10% TC; this high C content may have influenced glucose-derived CUE after 2 days of incubation. Moreover, soil pH can alter the composition of microbial communities and consequently affect CUE (Manzoni et al., 2012; Zheng et al., 2019). Rousk et al. (2011) reported that fungal growth is suppressed at pH_(H2O)_ > 5.5, whereas bacterial growth is limited at pH_(H2O)_ < 5.5. Keiblinger et al. (2010) reported that fungi induced higher CUE than bacteria, and fungal CUE was affected by C availability and bacterial CUE was limited by N and P availability, respectively. Therefore, it is possible that the microbial community composition was adapted to low pH conditions and the high C content of the soil, and these factors may have contributed to the marginal effect of P fertilization on glucose-derived CUE in the non-allophanic Andosol.

Jones et al. (2019) reported that low pH and associated exchangeable Al decrease CUE through metabolic shifts and greater C diversion into maintenance and catabolic processes, rather than through a reduction in the ability to use labile C. In our study, the amount of exchangeable Al was 0 meq kg^−1^ in the HP soil and 21.30 meq kg^−1^ in the LP soil. It is possible that the lower glucose-derived CUE in the LP soils after 20 days of incubation, when the applied C substrate was largely consumed (with a remaining fraction of 23.40–59.21 mg C kg^−1^soil), was due to the toxicity of exchangeable Al, as reflected in the differing amounts of Al^3+^, in addition to the low soil P level. Although Jones et al. (2019) focused on short-term effects (2–72 h), our study suggests that these factors may affect CUE over longer time scales than several hours. The absence of a difference in CUE between the HP and the LP soils on day 2 of incubation, however, may have been due to the influence of organic C availability, as mentioned in the previous paragraph. Therefore, the low CUE observed in the LP soils is likely attributable to C investment for P acquisition and the response to acid and Al stresses.

Our results showed lower primed CO_2_-C of added glucose C in non-allophanic Andosols compared to previous studies. In this current study, the values ranged from −1 to 13% in the non-allophanic Andosols over 20 days of incubation compared to values of 28 to 420% for other soil types (Supplementary Table S4) (Shahbaz et al., 2018; Mehnaz et al., 2019; Sawada et al., 2023). This comparison is valid as the values were calculated from data between 18 and 42 days of the entire incubation period. In contrast, the size of the MBC pool was comparable to or even larger than that in these studies (100–1,190 mg C kg^−1^ soil), suggesting a higher total microbial biomass, including potentially active microbes. These lower PEs in the non-allophanic Andosols were possibly due to the abundance of Al-organic matter complexes. The stability of SOM depends on Al-organic matter complexes and soil acidity (Miyazawa et al., 2013). These complexes promote the formation of soil aggregates and have also been suggested to physically suppress the microbial SOM decomposition (Matsui et al., 2016). Therefore, it is suggested that the low PEs reflect SOM stability in non-allophanic Andosols.

Our findings indicate that maintaining C stocks in non-allophanic Andosols depends not only on adequate long-term P fertilization but also on appropriate regulation of soil acidity. However, we acknowledge that our experiment did not directly represent the temporal Al toxicity effects on CUE, and we could not statistically separate the individual contributions of these overlapping factors to the observed responses. Future studies need to establish these causal relationships by using controlled manipulations of soil pH and by investigating the relationships between Al bioavailability and C dynamics at different soil P levels.

## 5 Conclusion

This study showed that long-term P fertilization strongly influences substrate-induced SOM decomposition in non-allophanic Andosols. The addition of glucose with N promoted positive PEs under low P conditions, while negative PEs occurred under high P conditions, with LP soils consistently showing greater PEs throughout incubation. The lack of effect on MBP and MBC: P in LP soils, together with greater CUE in HP soils after 20 days, indicates that microbial C allocation for P acquisition under P-limited conditions regulates substrate-induced SOM decomposition. These findings also suggest that soil acidity and exchangeable Al may interact with P availability to influence microbial C dynamics, although their independent effects remain to be clarified. Future research should elucidate the interactive effects of P availability, soil pH, and exchangeable Al on microbial C dynamics in non-allophanic Andosols.

## Data Availability

The raw data supporting the conclusions of this article will be made available by the authors, without undue reservation.
